# The role of university teaching staff members in cognitive awareness and raising the level of health protection, value, and moral of students through the COVID‐19 pandemic

**DOI:** 10.1002/pa.2332

**Published:** 2020-08-19

**Authors:** Amani M. Al‐Hosan, Nawal M. AlRajeh, Boshra A. Arnout

**Affiliations:** ^1^ Faculty of Education Princess Nourah bint Abdulrahman University Riyadh Saudi Arabia; ^2^ Department of Psychology Faculty of Education, King Khalid University Abha Saudi Arabia; ^3^ Department of Psychology Faculty of Arts, Zagazig University Zagazig Egypt

## Abstract

This study aimed to detect the role of teaching staff members in increasing university students' awareness, health protection, moral, and value aspects through the e‐learning, and to reveal the differences in the level of the teaching staff members about their roles due to the path of college, academic degree, and academic experience. To achieve these aims of the study, the descriptive method was applied. The study sample consisted of (101) teaching stuff member in health, scientific, and human specializations at Princess Nourah Bint Abdul Rahman University in Saudi Arabia. The researchers designed a questionnaire to collect the data that reflects the perceptions of teaching staff members about their cognitive, skill, health, and ethical roles toward responding to the COVID‐19 pandemic through distance education. The results indicated that there are high levels of teaching staff members' perception of their skill, health, and ethical responsibilities to raise students' awareness about the COVID‐19 pandemic, while the level of teaching staff members' perception of their cognitive responsibilities to raise students' awareness about the COVID‐19 pandemic was average. The findings indicated there are differences in the perception of the teaching staff members of their cognitive, skill, health, and moral value responsibilities to raise students' awareness about the COVID‐19.

## 1 INTRODUCTION AND THEORETICAL BACKGROUND

Educators and the researchers in the field of education and psychology agree that studying the cognitive components and preventive precautionary related to public health from epidemics such as COVID‐19 pandemic, which acquires the importance and priority of research due to its association with the public health system. This study came because of the scarcity of studies conducted on cognitive, health, and ethical aspects, and the lack of specialized literature, and as a try to face the inevitable human global fate that requires all countries in the world to initiate projects and develop strategies to increase health, psychological, and social awareness and combat dangerous pandemics that threaten human life, including COVID‐19, which is still harvested thousands of lives a day. According to the statistics of the World Health Organization in a number of developed countries until now, the statistics of injuries exceeded three and a half million worldwide, and the number of deaths exceeded two hundreds of thousands.

Arnout (2020) mentioned that COVID‐19 is a new strain that was discovered in 2019 and has not been previously identified in humans. Common signs of infection include respiratory symptoms, fever, cough, shortness of breath, and breathing difficulties. In more severe cases, infection can cause pneumonia, severe acute respiratory syndrome, kidney failure, and even death (WHO, 2020a). On March 11, 2020, the Director‐General of WHO declared the spate of infections caused by SARS‐CoV‐2 pandemic.

Since the beginning of February 2020, With the spread of COVID‐19 in the Chinese city of Wuhan and its transmission to Europe, America, and the Arab Gulf countries, including the Kingdom of Saudi Arabia, it was necessary for the responsible government sectors take all procedures to cope the infection by spreading a culture of preventive health and taking all precautionary procedures, such as stay at home.

Arnout et al. (2020) attempted to show that the current crisis of the spread of the COVID‐19 virus pandemic has swept the world, leaving infections in large numbers, and global health systems have collapsed with them, and large and advanced countries have not faced the challenges of confrontation. As of March 28, 2020, the Coronavirus (COVID‐19) pandemic had caused more than 1.6 billion children and young adults to drop out of education in 161 countries, nearly 80% of students enrolled in schools worldwide. This came at a time when we are already suffering from a global education crisis. There are many students in schools, but they do not receive the basic skills they need in working life.

There is no doubt that e‐learning is one of the scenarios that the countries of the world have resorted to facing the spread of the COVID‐19, and not disrupting the educational process. Distance education is one of the most important concepts and modern technologies for university education at all levels. Faculty members in universities have an important impact on their students by communicating the information needed for them in a correct and scientific manner.

Hence, in light of the COVID‐19 pandemic outbreak in the worldwide, and the importance of paying attention to the pivotal role of the faculty university members who has become today a major influence in enhancing the level of student health with the development of the social perception of health and its consideration of behavior and lifestyle, and with findings of studies which emphasized the enactment of public health policies and legislation, and providing students with the appropriate amount of information and preventive trends related to diseases and health problems, enabling them to protect themselves in a proper scientific manner.

Educational institutions and university education have an important role in building a health system for students, by promoting healthy and preventive life practices in their daily life. University is an integrated community in which many segments of society live or interact with it, such as students, teachers, administrative staff, and parents. Because health education has always targeted broad groups in order to generalize awareness, positive attitudes, and behaviors of health and life, the university is an effective and essential center for communicating awareness and education messages effectively to a large segment of society if it is best communicated in a systematic scientific manner. The promotion of community health through the university environment works to devote health education to students, by highlighting its components faculty members, educational curricula, and school activities, which work to spread health awareness among students, and inform them of the need to take care of their health, preserve them, and inform them of their physical conditions.

Hence, in cooperation with the great efforts and health care given by the Saudi government represented in the Ministry of Health in terms of health, field, digital, and community services and efforts to decrease the spread of the COVID‐19 and frame its scope. This study comes from an academic research team at Princess Nourah Bint Abdul Rahman University as a contribution to its educational role and the community to raise the level of positive perceptions and awareness of health, values and ethics to deal with the Corona virus among university teachers staff.

### 1.1 Preventive education and COVID‐19 pandemic

Studies in the health field emphasized that preventing against diseases is the best way to cope and respond to the disease before it occurs. Awareness is considered one of the most important educational processes, and an important means for the teacher to develop a university education system, especially in times of crisis. Without awareness of its various levels: cognitive, health, and values. Confusion and imbalance may occur within the educational environment, especially in times of crisis, which helps to make optimal use of resources, to achieve the desired aims, with the highest degree of efficiency, according to the e‐learning and distance learning system (Abdel Hai, 2006).

The spread of COVID‐19 has been declared a public health pandemic that causes international concern, and the disease has now spread to many countries and regions. While there is still much that we do not know about the virus that causes COVID‐19 disease, we know that it is spread through direct contact with the respiratory droplets of an infected person (which is caused by coughing or sneezing). Individuals can also become infected by coming into contact with contaminated surfaces of the virus and then touching their faces (eyes, nose, and mouth). As COVID‐19 continues to spread, it is important for communities to take action to prevent further infection, reduce the impact of the disease, and support procedures to control it (WHO, 2020b).

It is important to remember that COVID‐19 does not distinguish between borders and ethnic groups, nor between individuals in terms of disability, age or gender. Educational spaces must remain welcoming, respectful, and inclusive environments. The actions of schools and universities can prevent COVID‐19 from spreading through students.

### 1.2 The role of university teaching staff in COVID‐19 pandemic management

NBO (2020) found that faculty members can use common strategies in online learning, such as blogs that instruct students to use collaborative writing methods or story making content production such as word processing, spreadsheets, and so on. Forums discussion, online teaching and evaluation tools, mind mapping, and the use of interactive maps and graphs, through distance learning and in emergencies, crises, and epidemics, teachers should be innovative, creative, and flexible, providing opportunities for learners gain awareness and knowledge of health and ethical, they may not get in normal times and circumstances.

According to SAS (2020), the university teaching staff insight students by using technology effectively through timely feedback to achieve better learning for students; this is especially true in online learning environments when students are not able to ask questions as they usually do in a direct classroom environment. According to UNICEF (2020), students should understand age‐appropriate basic information about COVID‐19 disease, including its symptoms and complications, how it is transmitted, and how to prevent its transmission. Such as instructing pupils and students to be informed about COVID‐19 pandemic through reputable sources such as the National Ministry of Health, while directing students to use critical thinking in interacting with counterfeit information that may be circulated orally or over the internet.

About the role of educational institutions in the United States of America in coping COVID‐19 pandemic crisis, the Oklahoma (2020) conducted a study on the uses of e‐learning in times of crisis and epidemics, so you see virtual education as a form of distance learning, but it is far from the participation of students in educating them only if properly used. Many areas of Oklahoma, especially in rural communities, lack access to the internet.

It is important to note that distance learning does not require digital technology or communication. According to the Technology Capacity Survey conducted by OSDE, more than 25% of the regions will ensure learning, in whole or in part, through paper packages of educational materials distributed to students. OSDE provided a bunch of aid. The distance learning resources page of the OSDE website provides comprehensive assistance to the provinces to suit the needs of its students. In addition, OSDE has partnered with OETA TV to provide learning opportunities at home through quality daytime educational programming. According to him, the teacher can, in the absence of technological methods for distance learning to achieve moral, value, and cognitive awareness in times of crisis and epidemics, find alternatives available through social media or through traditional media through government or private television.

The study of the British Psychological Association (2020) about how we can strengthen the resilience and positive adjustment of teachers to cope COVID‐19 crisis. Results indicated that resilience is a process corresponding to an internal personality trait. It can change over time depending on the context or situation. Resilience can be more effectively channeled if it is the focus of society, especially within educational organizations, to support the resilience of teachers in times of crisis and epidemics.

On the other hand, the Hastings Center (2020) conducted a study to develop a framework for ethically sound work to achieve health education during the crisis, to achieve a balance between the duty of customer‐centered care, and focus on ethics for ethical practice under normal circumstances. With audience‐centered duties to support equal distribution of risks and social benefits, this study is designed to work on general health practice and guidance about COVID‐19. It aimed to help build ongoing discussions of predictable ethical issues arising from emergency care conditions by asking practical questions in public health emergencies, in situations of planning, protection, and guidance.

Hamedani, Haghani, and Kelishadi (2020) referred that considers continuing education, especially among students, is the best way to modify their life style, and controlling and improving factors affecting lifestyle make school education more effective, especially in times of epidemics. As same, in a study conducted by Noormah and Jainatul (2017), the results indicated that all teachers in the study preferred to use teaching‐centered teaching strategies, and few made an effort to integrate student‐centered teaching strategies in their lessons. The results also revealed that students were more involved in lessons that provided an offer to learn with experience and collaborative learning. The findings of this study benefit teachers, researchers, policy makers, to decision‐making process in the epidemiological situations.

Recently, Al‐Rabiaah et al. (2020) pointed to the corona viral respiratory syndrome in the Middle East, and the situation in the Kingdom of Saudi Arabia. This epidemic could put a great stress on students' learning and mental health. In the same context, the cross‐sectional study conducted by Asaad, El‐Sokkary, Aedh, Alzamanan, and Khalil (2019) in the Kingdom of Saudi Arabia aimed to study knowledge and attitude toward Corona Virus (MERs‐COV) among health practitioners. The results found that the medical students had better knowledge compared to students of other health colleges. These results highlight the necessity of designing educational courses on new emerging diseases, epidemiology, and infection control practices for health sciences students to prepare them to deal with these types of health emergencies.

The Community Health Center Guidance in Higher Education in 2020 conducted a study about COVID‐19, aimed to identify the role of schools in responding to COVID‐19, and the best way to prevent infection is to avoid exposure to the virus it causes, and learn more about the epidemic. The results indicated that the higher education institutions working with local health departments to reduce the spread of the COVID‐19 pandemic. Higher education efforts will help to ensure a healthy and safe environment for learning and work, and also provide accurate information about COVID‐19 to students and faculty members, to reduce the stigma associated with COVID‐19.

As for the World Health Organization (WHO, 2020a) told us we knew little about the causes of COVID‐19, but we know that it is transmitted through direct contact, whether from sick people spray, cough, and sneeze, and also from touching surfaces contaminated with the virus and then touching their faces.

### 1.3 Preventive education and COVID‐19 pandemic

The interest in preventive education stems from being an essential part in the lives of individuals in societies, as well as from the fact that its success in achieving its goals, has greatly changed the nature of the great losses that we observe in our lives, health, economy, and others. Al‐Qumaizi (2006) mentioned that the education and prevention are among the modern concepts that have emerged in the educational and scientific field, so linguistic dictionaries have not had a separate definition, while researchers find linguistic definitions of education and prevention. He defined the preventive education as the set of knowledge, skills, and attitudes that must be included in the content of the curricula and given to students in order to help them make the appropriate decisions to face problems, crises, and disasters in a correct way.

Al‐Qumaizi (2006) referred that researchers set a number of aspects and requirements for preventive education, these aspects could be divided into:

1. *Food and Health*: In this regard, students are taught and trained to apply the foundations of healthy nutrition and familiarity with the means of preparing and eating the food they need both physically and psychologically and persuading them of the benefits of food and its impact on the health of their bodies, as they are defined by the different ways of preserving foods and how to prevent food diseases, and it should also emphasize the importance of accustoming students on breakfast.
2. *Prevention of endemic and infectious diseases*: In it, students are provided with the appropriate amount of information and preventive trends related to diseases and health problems, which enables them to protect themselves from these diseases before they occur and face them in a proper scientific manner.
3. *Smoking prevention, drug use, addiction, and narcotic drugs*: These matters are considered one of the dangerous inputs that have occurred to our Arab and Islamic societies and all societies alike and the danger that has spread to our students in the early days of their lives. In preventive education, students are provided with the correct information and directions about the damages resulting from smoking, drug use, addiction, and narcotic drugs.
4. *Prevention of natural and industrial disasters and their risks*: It is a set of information, knowledge, and safety and security measures that must be given to students about natural or industrial disasters and their effects on the environment in order to help them protect themselves and their environment from their damages. In this aspect, students are also made aware of the importance of self‐control and lack of panic when catastrophes and disasters occur.
5. *Safety and Security*: In it, students are accustomed to practicing their activities inside the school without exposure to hazards or harming their school, and they are also introduced to how to prevent dangerous animals, the dangers of electricity, gases, fires, elevators, traffic accidents, transit, falls, and slip, as they are trained in first aid and how to prevent sharp and dangerous machines and materials, and chemical and flammable materials.

### 1.4 The importance of distance learning in COVID‐19 pandemic crises

The importance of distance learning emerges at a time of disasters which drives educational institutions to implement without planning, in order to overcome the educational crises due to disasters. The emergency transformation of distance education is the best practices in education during crises and disasters. The urgent transformation of distance education in light of the COVID‐19 epidemic that imposed on the world isolation and quarantine necessitated the rapid transformation of applying the distance education system to ensure the progress of the educational process and not stop it. There are a number of criteria and determinants that require achieving quality when applying the distance learning in crises, as follows:

1. Instilling societal feeling by encouraging and guiding students to introduce themselves and participate in virtual classes and educational platforms.
2. Explain how students use the learning materials available in digital environments helping them complete course activities to achieve learning goals.
3. Clarify the relationship of activities and duties with the objectives of the course set as well as clarify the evaluation methods.
4. Providing students with feedback times so that they can follow their progress in the course.

### 1.5 Criteria of the effective teaching in a new virtual environment

Al‐Hosan and Oyaid (2012) mentioned that to achieving the best mechanism for effective teaching to create a new virtual environment requires a number of criteria:

1. Using brief multimedia to interact with students.
2. Organizing the e‐course content to guide students during the learning period and assist them continuously to navigate through the course content every week.
3. Planning for active learning using the course tools to facilitate the interaction of students.
4. Providing students with appropriate information to protect their personal information and their privacy by providing tools or suggestions through the course.
5. Providing the necessary licenses for the tools used in the e‐ course.
6. Providing clear directions and instructions for the e‐learning environment in a digital format with a clear definition of the components of the course and what the student must do for a correct start.
7. Providing students directly with sudden changes related to course policies.
8. Clarify quick and easy ways for students to find academic resources and resources and support services remotely.
9. Providing students with instructions and evaluation mechanism.
10. Determine where students can get immediate support for the course and inform them in advance about the techniques that they will need to obtain.
11. Explain to students how they access the educational institution's services and provide the help they need to access the digital course tools.

### 1.6 E‐learning platforms and COVID‐19 pandemic crisis

UNESCO noted that the wealth of digital educational resources has made new demands on education systems and institutions, which include developing innovative curricula, alternative educational paths, and innovative qualitative teaching methods, all of which can be facilitated via the Internet, distance education, and short skills‐based courses. The organization has developed a set of programs that help with distance learning, including the “Black Board” application, which is an application that depends on designing courses, assignments, tasks, preparing online tests and correcting them, and communicating with students through a virtual environment as well as by applications downloaded via smart phones.

Also, the “Edmodo” platform, which is a free social platform that provides teachers and students with a safe environment for communication and cooperation, and the exchange of educational content and digital applications, in addition to homework, grades, and discussions. The “Adrak” application, which is concerned with teaching Arabic online, and the Google Classroom application, which facilitates communication between teachers and students, whether inside or outside the school. Likewise, seesaw, a digital application that helps students document what they learn in school and share it with teachers, parents, and classmates, and even the world, and Mind spark, which is based on an adaptive online learning system, helps students practice and learn mathematics.

There are many e‐learning applications available on the Internet, some of which are free of charge, and video display platforms such as YouTube can be used, for example, as e‐learning platforms by converting lessons into videos that any student can view from anywhere in the world. But what distinguishes the specialized platforms in e‐learning is providing the capabilities of direct and indirect communication between the student and the teacher, in a way that helps to enhance the educational process with its basic components without any disruption leading to the failure to complete the education or teaching system properly.

There are very famous platforms in the field of e‐learning, such as the Moodle platform, which is the most famous in the world in the field of online education and provides many tools, whether for the teacher, school, or student to implement the educational process in an integrated manner, and this platform is completely free and open source, and can be installed once on any server. To provide a practical and quick solution to convert the educational system into an electronic system immediately, it is available in many languages, including Arabic. What is certain here is that there are many modern technological solutions in the field of online education and online teaching, but for the success of these solutions, those who implement them must be students of education technology, that is, they simply must combine understanding the teaching and education system, and are fully aware of the components of those The system, as they must understand this technology and its tools and capabilities to adapt those capabilities to complete the educational system successfully.

Any lack of understanding between these two sides will lead to a disruption in the educational process necessarily, and to the failure of the e‐learning or online education system, so it is necessary to resort to reliable bodies in implementing e‐learning operations to supervise in a scientific and systematic way the training of teachers and students and those in charge of the educational process to switch from traditional education or the direct pattern to online education online without the educational system being affected at all, most of the achievement and provision of all components of that educational system, but via the internet. Resorting to specialized agencies in the field of training and electronic education is the ideal choice for schools, bodies, and governments looking to invest that technology in the transition toward online education in times of crisis and the spread of epidemics to overcome those crises without stopping the educational system.

In Saudi Arabia, the Ministry of Education has succeeded in interacting with the COVID‐19 crisis significantly, and the amount of effort and meetings and the mobilization of all human and technical capabilities in continuing distance education, after the launch of the distance learning system and good use of the future portal, teachers and faculty members gave their experiences to continue the educational process and facilitate educational alternatives for male and female students and to complete their educational trip (Arnout, 2020).

## 2 OBJECTIVE

The purpose of this study is to provide practical guidance for safe operations through prevention, early detection, and disease control in universities. This study seeks to detect the level of university teaching staff's perception about their role in cognitive awareness, health protection, and the promotion of moral value aspects among students toward the COVID‐19 pandemic as a constraint via the e‐learning system. As well as, to reveal the differences in level of the university teaching staff about their roles (due to the path of the college, academic degree, and academic experience).

## 3 METHODOLOGY

### 3.1 Method

The current study methodology is based on examining university teaching staff's perceptions and opinions regarding their cognitive, skill, health, and ethical roles toward addressing the COVID‐19 pandemic. The study applied the descriptive method, which achieves its aims to identify university teacher members' perception toward their educational and educational roles toward responding to the COVID pandemic.

### 3.2 Participants

**FIGURE 1:**
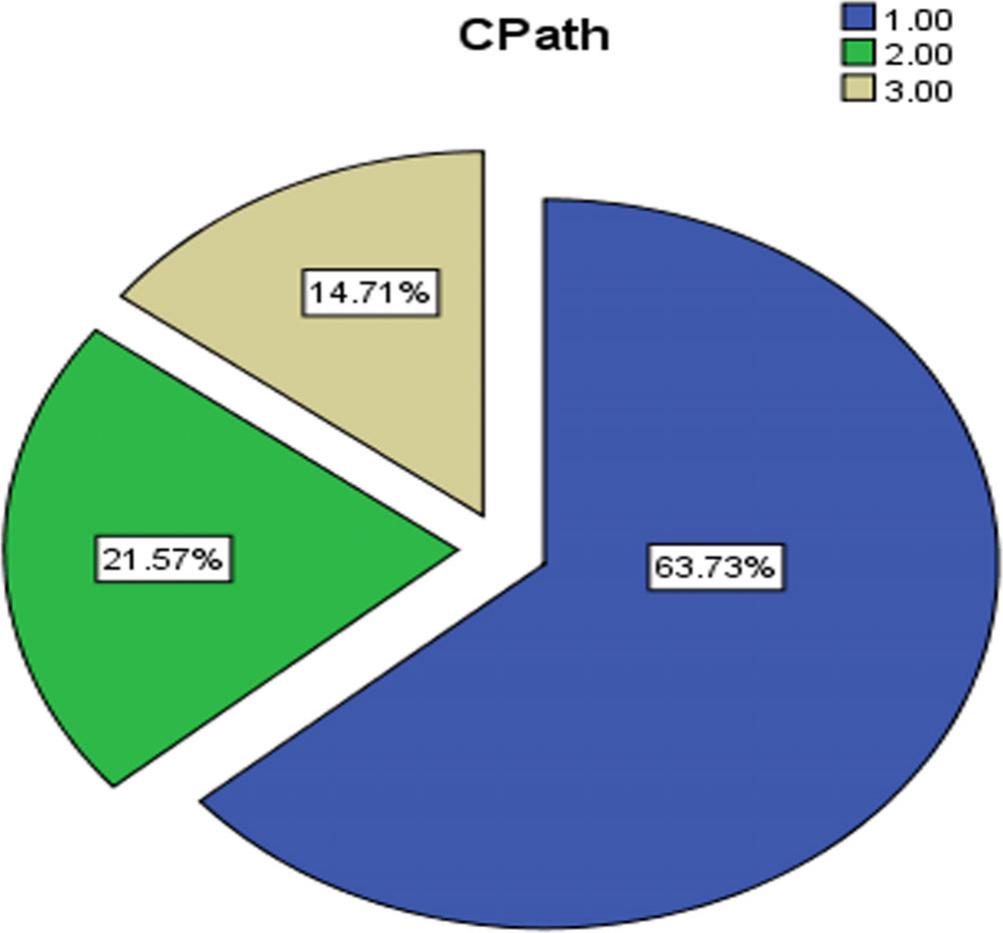
Study sample distribution according to the college pathway

**FIGURE 2:**
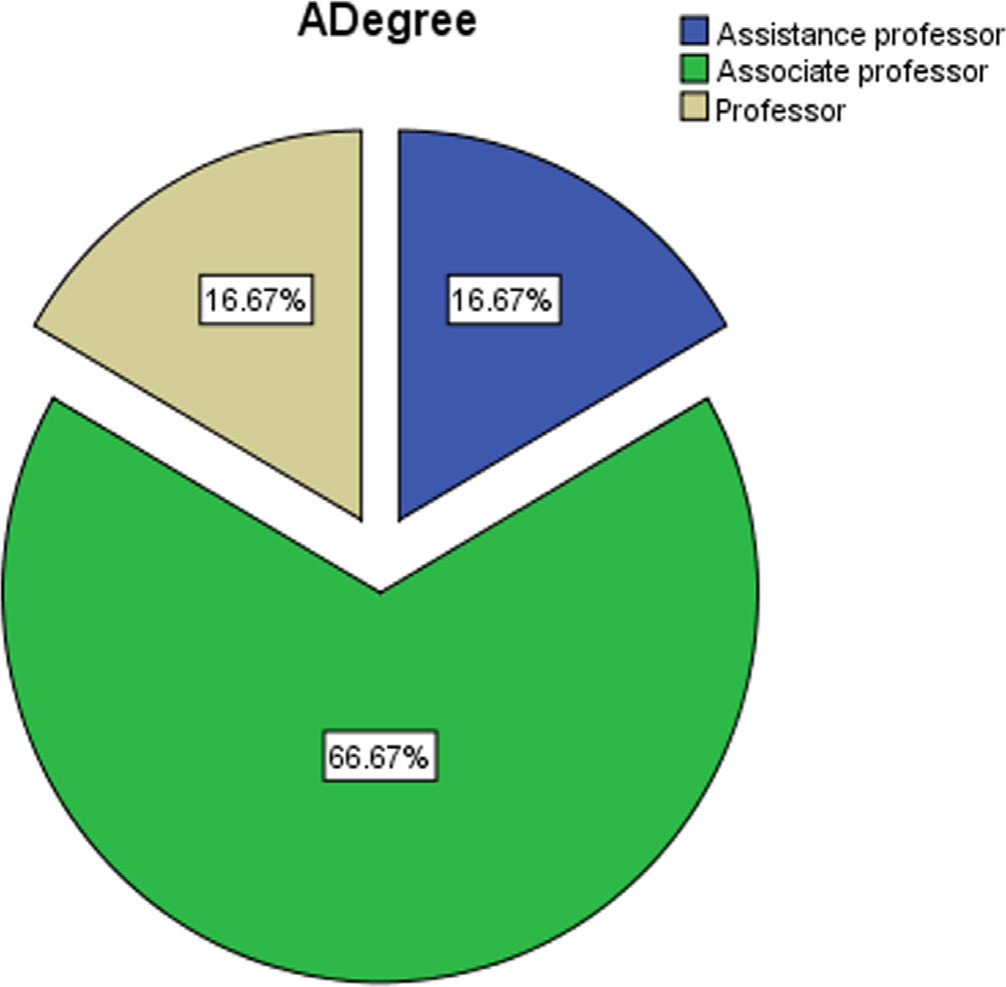
Study sample distribution according to the academic degree

**FIGURE 3:**
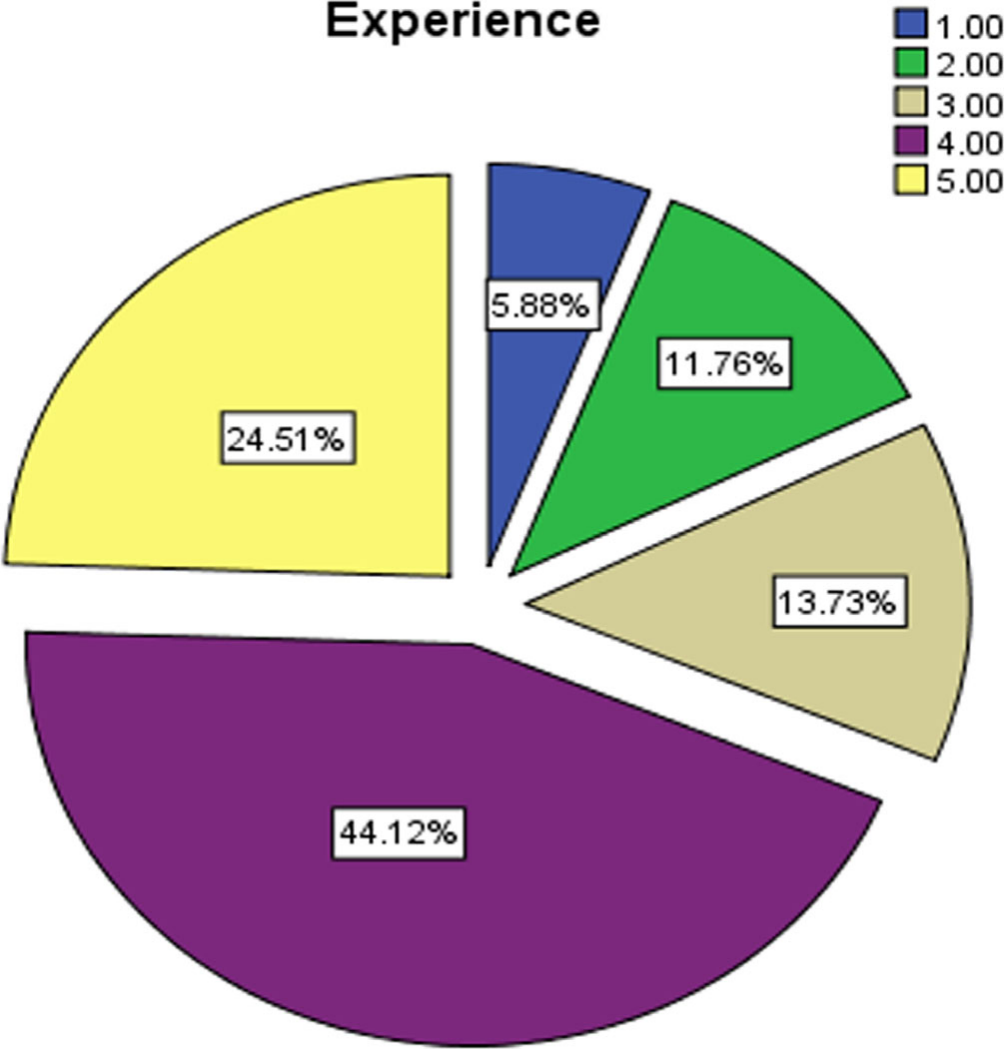
Study sample distribution according to academic experience

The current study community consisted of the faculty members at Princess Nora Bint Abdul Rahman University in the Kingdom of Saudi Arabia during the academic year (2019–2020). A random sample was chosen from the study population, which consisted of (n = 101) teacher university member. They were divided into sub‐groups according to the path of the college, academic degree, and academic experience (see Figures 1, 2, 3).

### 3.3 Instruments

*The perception of a university member staff roles toward COVID‐19 pandemic questionnaire (PRQ‐54)*:

The perception of a university member staff roles toward COVID‐19 pandemic questionnaire through distance education includes 54 items, was prepared by the researchers in this study. The PRQ‐54 consisted of four, that are: cognitive, skillful, health, and moral perceptions of the COVID‐19 pandemic. The individual responds with a 5‐point Likert scale (never = 1 to always = 5). The validity and reliability of the scale were verified. The results showed in Tables 1, 2, 3 indicate that the PRQ‐54 is validated and reliable.

**TABLE 1:** Correlations between items and dimensions of PRQ‐45

Item	r	Item	r	Item	r	Item	r	Item	r	Item	r	Item	r	Item	r
1	.570**	8	.866**	15	.562**	22	.851**	29	.923**	36	.875**	43	.854**	50	.791**
2	.746**	9	.897**	16	.854**	23	.879**	30	.922**	37	.864**	44	.869**	51	.875**
3	.819**	10	.916**	17	.706**	24	.892**	31	.950**	38	.785**	45	.841**	52	.823**
4	.821**	11	.749**	18	.811**	25	.886**	32	.931**	39	.831**	46	.807**	53	.890**
5	.836**	12	.776**	19	.891**	26	.872**	33	.952**	40	.791**	47	.879**	54	782**
5	.840**	13	.801**	20	.874**	27	.889**	34	.946**	41	.684**	48	.853**
7	.836**	14	.826**	21	.896**	28	.872**	35	.889**	42	.863**	49	.879**

**TABLE 2:** Correlation matrix between dimension and the total score of the PRQ‐45

		Total score	Dis1	Dis2	Dis3	Dis4
Total	Pearson correlation	1	.952**	.985**	.944**	.952**
	Sig. (2‐tailed)		.000	.000	.000	.000
	N	102	102	102	102	102
Dis1	Pearson correlation	.952**	1	.927**	.849**	.873**
	Sig. (2‐tailed)	.000		.000	.000	.000
	N	102	102	102	102	102
Dis2	Pearson correlation	.985**	.927**	1	.939**	.904**
	Sig. (2‐tailed)	.000	.000		.000	.000
	N	102	102	102	102	102
Dis3	Pearson correlation	.944**	.849**	.939**	1	.862**
	Sig. (2‐tailed)	.000	.000	.000		.000
	N	102	102	102	102	102
Dis4	Pearson correlation	.952**	.873**	.904**	.862**	1
	Sig. (2‐tailed)	.000	.000	.000	.000	
	N	102	102	102	102	102

**Correlation is significant at the 0.01 level (2‐tailed).

**TABLE 3:** Cronbach's Alpha coefficients for the PRQ‐45

Dimensions	Alpha‐ Cronbach
Dis1	.958
Dis 2	.980
Dis 3	.976
Dis 4	.972
Total score	.991

From the results showed in Tables 1 and 2, we noticed that the PRQ‐54 items are related significantly with their dimensions. As well as, the dimensions also correlated significantly with the total score of the questionnaire, that mean this scale is characterized by internal consistency.

The findings showed in Table 3 about the Cronbach's Alpha coefficients for the PRQ‐54, indicated that the questionnaire is reliable.

### 3.4 Research design

To detect the level of university teaching staff perception about their role in the cognitive awareness, health protection, and the promotion of ethical value aspects among students during the COVID‐19 pandemic as a constraint via the e‐learning system. As well as, to reveal the differences in the level of the teacher members about their roles due to the path of the college, academic degree, and academic experience, a survey descriptive design was used in this study. The perception of a university member staff roles toward COVID‐19 pandemic questionnaire (PRQ‐54) has been applied to a random sample from faculty members at Princess Nora Bint Abdul Rahman University in the Kingdom of Saudi Arabia during the academic year (2019–2020) have responded to the questionnaire and sent it back to the researchers.

### 3.5 Data analysis

The data collected from the study sample were analyzed by using SPSS 25.0, and mean, standard deviation, one sample *t*‐test, and one‐way ANOVA were calculated

## 4 RESULTS

### 4.1 The results about the level of perception of faculty members toward their responsibilities to raise the cognitive, skill, health, and moral value awareness among students toward COVID‐19 through the distance learning

To determine the level of the perception of faculty members toward their responsibilities, *t*‐test value of one sample was calculated to detect the differences between the hypothetical mean and the mean scores of the individuals in the perception of faculty members toward their responsibilities. The results are shown in Table 4.

**TABLE 4:** One‐sample statistics

Dimensions	M	SD	t	Sig. (2‐tailed)
dis1	50.059	14.078	1.477	.143
dis2	64.412	21.203	2.101	.038
dis3	30.186	10.347	3.110	.002
dis4	61.686	16.523	6.532	.000
Total score	206.343	59.796	8.503	.000

The results shown in Table 4 indicate that there are high levels of the perception of faculty members at Princess Nora Bint Abdul Rahman University in the Kingdom of Saudi Arabia about their responsibilities to raise the skill, health, moral values, and total score (of their responsibilities in raising the students' awareness toward COVID‐19 through the distance learning). The differences for one sample (2.101, 3.110, 6.532, 8.503) were statistically significant at the level (0. 038, 0.002, 0.000, 0.000), respectively. While the level of the university teaching staff's perception toward their responsibilities to raise the cognitive students' awareness toward COVID‐19 through the distance learning was average, the differences for one sample were not statistically significant (t = 1.477 sig.143).

### 4.2 The results about the differences in the responses of the study sample's perception toward their responsibilities to raise students' awareness about the COVID‐19 due to the path of the college

One‐way ANOVA calculated to detect the differences in the responses of the study sample's perception toward their responsibilities to raise students' awareness about the COVID‐19. The findings are shown in Tables 5 and 6.

**TABLE 5:** ANOVA

		Sum of squares	df	Mean square	F	Sig.
D1	Between groups	2,765.983	2	1,382.991	7.936	.001
	Within groups	17,251.664	99	174.259		
	Total	20,017.647	101			
D2	Between groups	5,146.024	2	2,573.012	6.327	.003
	Within groups	40,258.682	99	406.653		
	Total	45,404.706	101			
D3	Between groups	738.393	2	369.197	3.628	.030
	Within groups	10,075.068	99	101.768		
	Total	10,813.461	101			
D4	Between groups	1,986.194	2	993.097	3.842	.025
	Within groups	25,587.767	99	258.462		
	Total	27,573.961	101			
Total	Between groups	36,532.818	2	18,266.409	5.571	.005
	Within groups	324,598.172	99	3,278.769		
	Total	361,130.990	101			

**TABLE 6:** The results of Scheffe test for differences due to the path of the college

Dependent variable	(I) College path	(J) College path	Mean difference (I‐J)	Std. error	Sig.	95% confidence interval	
						Lower bound	Upper bound
Dis1	1.00	2.00	‐.01678‐	3.25604	1.000	‐8.1089‐	8.0753
		3.00	‐14.70769‐*	3.78130	.001	‐24.1052‐	‐5.3102‐
	2.00	1.00	.01678	3.25604	1.000	‐8.0753‐	8.1089
		3.00	‐14.69091‐*	4.42020	.005	‐25.6762‐	‐3.7056‐
	3.00	1.00	14.70769*	3.78130	.001	5.3102	24.1052
		2.00	14.69091*	4.42020	.005	3.7056	25.6762
Dis2	1.00	2.00	‐5.51189‐	4.97398	.543	‐17.8735‐	6.8497
		3.00	‐20.45128‐*	5.77637	.003	‐34.8070‐	‐6.0955‐
	2.00	1.00	5.51189	4.97398	.543	‐6.8497‐	17.8735
		3.00	‐14.93939‐	6.75237	.092	‐31.7207‐	1.8419
	3.00	1.00	20.45128*	5.77637	.003	6.0955	34.8070
		2.00	14.93939	6.75237	.092	‐1.8419‐	31.7207
Dis3	1.00	2.00	‐3.46434‐	2.48827	.383	‐9.6483‐	2.7197
		3.00	‐7.37949‐*	2.88968	.043	‐14.5611‐	‐.1979‐
	2.00	1.00	3.46434	2.48827	.383	‐2.7197‐	9.6483
		3.00	‐3.91515‐	3.37793	.513	‐12.3102‐	4.4798
	3.00	1.00	7.37949*	2.88968	.043	.1979	14.5611
		2.00	3.91515	3.37793	.513	‐4.4798‐	12.3102
Dis4	1.00	2.00	1.03217	3.96543	.967	‐8.8229‐	10.8873
		3.00	‐12.14359‐*	4.60512	.035	‐23.5885‐	‐.6987‐
	2.00	1.00	‐1.03217‐	3.96543	.967	‐10.8873‐	8.8229
		3.00	‐13.17576‐	5.38322	.055	‐26.5544‐	.2029
	3.00	1.00	12.14359*	4.60512	.035	.6987	23.5885
		2.00	13.17576	5.38322	.055	‐.2029‐	26.5544
Total	1.00	2.00	‐7.96084‐	14.12365	.853	‐43.0617‐	27.1400
		3.00	‐54.68205‐*	16.40205	.005	‐95.4453‐	‐13.9188‐
	2.00	1.00	7.96084	14.12365	.853	‐27.1400‐	43.0617
		3.00	‐46.72121‐	19.17341	.056	‐94.3720‐	.9295
	3.00	1.00	54.68205*	16.40205	.005	13.9188	95.4453
		2.00	46.72121	19.17341	.056	‐.9295‐	94.3720

*The mean difference is significant at the 0.05 level.

The results showed in Table 5 indicated that there are significant statistical differences due to the path of the college in the responses of the study sample's perception toward their responsibilities to raise students' awareness about the COVID‐19 pandemic. To determine the direction of these differences, a Scheffe test was used (see results in Table 6).

As the results indicate (see Table 6) that the differences in the study sample's perception about their responsibilities to raise students cognitive, skill, health, moral value, and total score awareness about the COVID‐19 due to the path of the college, these differences were in the favor of the scientific college (see Tables 5 and 6).

### 4.3 The results about the differences in the responses of the study sample perception about their responsibilities to raise students' awareness about the COVID‐19 due to the academic degree

One‐way ANOVA calculated to detect the differences in the responses of the study sample perception toward their responsibilities to raise students' awareness about the Corona virus due to the academic degree. The findings are shown in Table 7.

**TABLE 7:** ANOVA

		Sum of squares	Df	Mean square	F	Sig.
Dis1	Between groups	497.721	2	248.860	1.262	.288
	Within groups	19,519.926	99	197.171		
	Total	20,017.647	101			
Dis2	Between groups	1,124.471	2	562.235	1.257	.289
	Within groups	44,280.235	99	447.275		
	Total	45,404.706	101			
Dis3	Between groups	66.637	2	33.319	.307	.736
	Within groups	10,746.824	99	108.554		
	Total	10,813.461	101			
Dis4	Between groups	216.623	2	108.311	.392	.677
	Within groups	27,357.338	99	276.337		
	Total	27,573.961	101			
Total	Between groups	6,021.225	2	3,010.613	.839	.435
	Within groups	355,109.765	99	3,586.967		
	Total	361,130.990	101			

The results showed in Table 7 indicated that there are no differences due to the academic degree in the responses of the study sample perception toward their responsibilities to raise students' awareness about the COVID‐19 pandemic.

### 4.4 The results about the differences in the study sample's perception toward their responsibilities to raise students' awareness about the COVID‐19 pandemic due to academic experience

One‐way ANOVA calculated to detect the differences in the responses of the study sample's perception toward their responsibilities to raise students' awareness about the COVID‐19 pandemic. The findings are shown in Table 8.

**TABLE 8:** ANOVA

		Sum of squares	Df	Mean square	F	Sig.
Dis1	Between groups	585.839	4	146.460	.731	.573
	Within groups	19,431.808	97	200.328		
	Total	20,017.647	101			
Dis2	Between groups	1,048.504	4	262.126	.573	.683
	Within groups	44,356.202	97	457.280		
	Total	45,404.706	101			
Dis3	Between groups	129.262	4	32.316	.293	.882
	Within groups	10,684.199	97	110.146		
	Total	10,813.461	101			
Dis4	Between groups	712.942	4	178.236	.644	.633
	Within groups	26,861.019	97	276.918		
	Total	27,573.961	101			
Total	Between groups	8,291.115	4	2,072.779	.570	.685
	Within groups	352,839.875	97	3,637.524		
	Total	361,130.990	101			

The results showed in Table 8 indicated that there are no differences due to the academic experiences in the responses of the study sample's perception toward their responsibilities to raise students' awareness about the COVID‐19 pandemic due to academic experience.

## 5 DISCUSSION

The results of the current study are consistent with what it confirmed by (SAS, 2020) that the teacher is increasing their students' awareness through using the technology effectively, including distance learning. It also agrees with what UNICEF (2020) called for, to educate students and provide them with basic information about COVID‐19, its implications, methods of transmission, and how to prevent its transmission. UNICEF also called for critical thinking among students in interacting with false information about COVID‐19 being circulated.

Therefore, the study that conducted by Jacquie, Bay, and Kamran (2017) reviewed the increasing global burden of communicable diseases (NCDs) and raised awareness of the need to develop primary risk prevention programs. The aims of facilitating long‐term behavior change in children and adolescents that can reduce the risk of these diseases have been established in school and university programs to improve the behavior of children and adolescents. These programs should be based on multiple branches of science, with the benefit of experience, thus we must highlight the role of teachers in developing students' awareness from COVID‐19.

The findings of the current study have revealed the high level of awareness of teachers at Princess Nourah Bint Abdul Rahman University about their role in educating students cognitive, health, skill, and value on COVID‐19, this is due to increase the awareness of officials and decision‐makers in the Kingdom of Saudi Arabia about the importance of educational institutions and their contribution to educating students to reduce injury rates, and this is what the results of Al‐Rabiaah et al. (2020) study showed that the death rate in the Kingdom of Saudi Arabia is the lowest compared to neighboring countries.

The findings of the current study indicated the important role of distance learning in increase the students' awareness about COVID‐19, and this was confirmed by the results of the OSDE study (2020) of the importance of e‐learning via distance in developing moral, value, and cognitive awareness in times of crises and epidemics. Likewise, the study conducted by the Emergency Center and Times of Crisis (TA, 2015) concluded that teachers have a prominent role in educating students in times of crises and disasters. As well as, the British Psychological Association (BPS, 2020) has emphasized support for teachers' resilience in times of crisis and epidemics. Noormah and Jainatul (2017) and Allen et al. (2002) emphasized the importance of the teachers role in educating students in times of crisis using teacher‐centered teaching strategies.

Recently, the Sahu's (2019) study recommended that the distance learning be invested in times of crisis and disaster. In the same context, the World Health Organization (WHO, 2019) called for schools, universities, and school health services to play an important role in educating students toward healthy lifestyles.

Therefore, Asaad et al. (2019) recommended the need to design educational courses on new emerging diseases and improve students' cognitive and awareness. More recently, the results of the CDC Center (2020) and Singh et al. (2017) recommended the importance of education, teacher, and higher education efforts to reduce the spread of the COVID‐19 by providing them with healthy health information and to reduce the stigma associated with COVID‐19.

In order for university faculty members to be able to protect students, they must provide a set of cognitive, skills, and attitudes via distance learning platforms in order to help them make appropriate decisions to properly address problems, crises, and disasters, including the COVID‐19 pandemic.

These findings confirm that the urgent transformation of distance learning in light of the global epidemic COVID‐19, which imposed on the world distance and quarantine, necessitated the rapid transformation of employing the distance learning system not only to ensure the progress of the educational process but also to educate students cognitive, skill, health, and values about this pandemic is as a protection for students from the risk of contracting this deadly virus, and here the role of universities and the teaching staff in activating this preventive and developmental role through distance learning platforms along with their educational role in the academic curricula is evident. What distinguishes the specialized platforms in e‐learning is the provision of the capabilities of direct and indirect communication between the student and their teachers, in a way that helps to enhance the educational process with its basic components without any disruption leading to the failure to complete the education or teaching system properly.

## 6 FUTURE DIRECTION AND RECOMMENDATIONS

The results of this study will direct the future studies on the effectiveness of using teacher‐based learning strategies to raise students' awareness of cognitive, skills, health, and moral values about COVID‐19, as well as on the effectiveness of distance learning in increasing student awareness about the effects and symptoms of COVID‐19. Directing future studies to prepare online programs and courses remotely and their effectiveness in health and preventive education for students.

As well as, the need to pay attention to the prevention of endemic and infectious epidemic diseases, students are provided with the appropriate amount of information and preventive attitudes related to diseases and health problems, enabling them to protect themselves from these diseases before occur and face them in a proper scientific manner, as it explains to them the importance of vaccination against diseases and the importance extermination of diseases that carry diseases and the importance of visiting a doctor when signs and symptoms of COVID‐19 appear.

In the same direction, we recommend the development of an electronic course on preventive education, which students study at different stages of education, so that this course selects topics that are appropriate to the level of each stage, as well as the conditions and needs of society and the environment in which students live in light of the need of COVID‐19. Also, the inclusion of the items and aspects of preventive education in the curricula by using the bridging strategy, and linking what students study with the situations and problems they face in their daily lives due to the outbreak of the COVID‐19 pandemic as one of the epidemics of the 21st century.

In light of the current study results, the researchers recommend several procedural recommendations, including the rehabilitation and training of faculty members on blackboard systems for e‐learning remotely to activate and use them effectively in crises. And also, we stressed on the use of learning materials and software available in digital environments for distance learning systems during crises. We also recommend employing effective and supportive strategies for the success of remote e‐learning in raising the level of cognitive, health, and moral value awareness. And we recommend the necessity of activating the e‐learning platforms, which include developing innovative curricula, study programs and alternative educational paths. In addition, activating development partnerships with companies specialized in the field of training and e‐learning to invest their experiences in a complete transformation toward e‐learning in crises. Also, setting up studied plans to manage e‐learning in times of crisis and the spread of epidemics, in order to ensure the continuity of the educational system in an efficient and successful manner.

## 7 CONCLUSION

In light of the study findings, we can say that it is necessary to increase the aspects of protection from epidemics, natural, and health disasters, by providing a set of information, knowledg,e and safety and security procedures that must be given to students about epidemics and infectious diseases, natural or industrial disasters, and their effects on them and the environment in order to help them protect themselves and their environment from damages, as is done in this regard, students are made aware of the importance of self‐control and not being dismayed when disasters, disasters, and epidemics occur.

## 8 LIMITATIONS

This study, despite its important results, has important applications, but it suffers from limitations. In terms of methodology, the reader should bear in mind that the study was based on the descriptive method, and the study sample was also small and restricted to university teaching staff at Princess Nora Bint Abdul Rahman University, but not to other universities. It also suffers from a third limitation, which is that it was concerned with revealing the role of teaching staff in universities only without studying teachers in the schools. For these reasons, we need future studies that apply other methodology, such as semi‐experimental and comparative causal. We also need other studies aim to revealing the role of teachers in schools in developing students' awareness about COVID‐19, and studying the degree of awareness of faculty members about developing student awareness through distance education about COVID‐19 disease and its effects and symptoms.

## CONFLICT OF INTEREST

The authors of this manuscript declare that they have no conflict of interests.

## ETHICS STATEMENT

The authors of this manuscript have complied with ethical principles in their treatment of individuals participating in the research policy described in the manuscript.

## Data Availability

All data underpin this study found in this manuscript, there are no any additional data.

## References

[pa2332-bib-0022] Abdel Hai, R. (2006). Educational planning, its importance, justifications and foundations. Cairo: Dar Al‐Wafaa for Printing and Publishing.

[pa2332-bib-0001] Al‐Hosan, A. , & Oyaid, A. (2012). Towards identifying quality assurance standards in virtual learning environments for science education. Pertanika Journal of Social Sciences & Humanities, 20(3), 797–828.

[pa2332-bib-0002] Allen, M. , Burt, K. , Bryan, E. , Carter, D. , Orsi, R. , & Durkan, L. (2002). School counselors' preparation for and participation in crisis intervention. Professional School Counseling, 6, 96–102.

[pa2332-bib-0003] Al‐Qumaizi, H. (2006). Preventive education. Al‐Khafji Journal, 36(2), 14–16.

[pa2332-bib-0004] Al‐Rabiaah, A. , Temsah, M. , Al‐Eyadhy, A. , Hasan, G. , Al‐Zamil, F. , Al‐Subaie, S. , … Somily, A. (2020). Middle east respiratory syndrome‐corona virus (MERS‐CoV) associated stress among medical students at a university teaching hospital in Saudi Arabia. Journal of Infection and Public Health, 13(5), 687–691. 10.1016/j.jiph.2020.01.005 32001194 PMC7102651

[pa2332-bib-0005] Arnout, B. (2020). COVID‐19 pandemic crisis: And the new face of the world. Germany: Scholars Press.

[pa2332-bib-0006] Arnout, B. , Al‐Dabbagh, Z. , Al Eid, N. , Al Eid, M. , Al‐Musaibeh, S. , Al‐Miqtiq, M. , & Al‐Zeyad, G. (2020). The effects of corona virus (COVID‐19) outbreak on the individuals' mental health and on the decision‐makers: A comparative epidemiological study. International Journal of Medical Research & Health Sciences, 9(3), 26–47.

[pa2332-bib-0007] Asaad, A. , El‐Sokkary, R. , Aedh, A. , Alzamanan, M. , & Khalil, F. (2019). Exploring knowledge and attitude toward middle east respiratory syndrome‐coronavirus (MERS‐CoV) among university health colleges' students, Saudi Arabia: A cross‐ sectional study. American Journal of Infectious Diseases and Microbiology, 15, 37–43. 10.3844/ajidsp.2019

[pa2332-bib-0008] British Psychological Association BPS (2020), Teacher resilience during coronavirus school closures. Retrieved from https://www.bps.org.uk/sites/www.bps.org.uk/files/Member%20Networks/Divisions/DECP/Teacher%20resilience%20during%20coronavirus%20school%20closures.pdf

[pa2332-bib-0024] CDC center (2020). Ethical Framework for Health Care Institutions Responding to Novel Coronavirus SARS‐CoV‐2 (COVID‐19) Guidelines for Institutional Ethics Services Responding to COVID‐19 Managing Uncertainty, Safeguarding Communities, Guiding Practice. https://snlg.iss.it/wp-content/uploads/2020/03/AA-Hastings-Center-Covid-Framework-2020.pdf

[pa2332-bib-0009] Emergency Center and Times of Crisis Center TA (2015). *Supporting continuity of learning and education*. Retrieved from https://rems.ed.gov/docs/Supporting_Continuity_of_learning_and_education.pdf.

[pa2332-bib-0010] Hamedani, Z. , Haghani, F. , & Kelishadi, R. (2020). Strategies to non‐communicable diseases prevention improvement from the viewpoints of students in Isfahan: A qualitative research. Journal of Education Health Promotion, 29(8), 232. 10.4103/jehp.jehp_218_19.eCollection PMC6905281 31867396

[pa2332-bib-0011] International Baccalaureate Organization (2020). Online learning, teaching and education continuity planning for schools. Retrieved from https://www.ibo.org/globalassets/news-assets/coronavirus/online-learning-continuity-planning-en.pdf

[pa2332-bib-0012] Jacquie, L. , Bay, R. , & Kamran, S. (2017). School‐based primary NCD risk reduction: Education and public health perspectives. Health Promotion International, 32, 369–379. 10.1093/heapro/daw096 28011654

[pa2332-bib-0013] Noormah, A. , & Jainatul, H. (2017). Teaching strategies to raise awareness of non‐communicable diseases in secondary schools in Brunei Darussalam. Proceeding of the 3rd International Conference on Education, 3, 77–90. 10.17501/icedu.2017.3109

[pa2332-bib-0014] OKLAHOMA (2020) https://sde.ok.gov/sites/default/files/FAQS%20FOR%20PUBLIC%20SCHOOLS%20-%20COVID-19.pdf.

[pa2332-bib-0015] Sahu, P. (2019). *Closure of Universities Due to Coronavirus Disease 2019 (COVID‐19): Impact on Education and Mental Health of Students and Academic Staff, Medical Education and Simulation*. Centre for Medical Sciences Education, The University of the West Indies, St. Augustine, TTO. 10.7759/cureus.7541 PMC7198094 32377489

[pa2332-bib-0016] SAS (2020). *Distance learning plan*. Retrieved from https://powerschool.saschina.org/public/HSPX1415.pdf.

[pa2332-bib-0017] Singh, A. , Bassi, S. , Nazar, G. , Park, S. , Kinra, S. , & Arora, M. (2017). Impact of school policies on non‐communicable disease risk factors – A systematic review. BMC Public Health, 17, 292 (2017). 10.1186/s12889-017-4201-3 28376833 PMC5379668

[pa2332-bib-0018] The Hastings Center, PUBLIC SCHOOL (2020). *Ethical framework for health care institutions responding to novel Coronavirus SARS‐CoV‐2 (COVID‐19) Guidelines for Institutional Ethics Services Responding to COVID‐19*. Retrieved from https://snlg.iss.it/wp-content/uploads/2020/03/AA-Hastings-Center-Covid-Framework-2020.pdf.

[pa2332-bib-0023] UNESCO (2020). Statement on COVID‐19: ethical considerations from a global perspective. https://unesdoc.unesco.org/ark:/48223/pf000037311

[pa2332-bib-0019] World Health Organization (2019). WHO guidelines for the health sector response to child maltreatment*. Technical Report*. Geneva: WHO. Retrieved from https://www.who.int/publications-detail/whoguidelines-for-the-health-sector-response-to-child-maltreatment.

[pa2332-bib-0020] World Health Organization (2020a). *WHO Awareness messages to prevent and control COVID‐19 disease in schools*.

[pa2332-bib-0021] World Health Organization (2020b). *WHO Key Messages and Actions for COVID‐19 Prevention and Control in Schools*. Retrieved from https://www.who.int/docs/default-source/coronaviruse/key-messages-and-actions-for-covid-19-prevention-and-control-in-schools-march-2020.pdf?sfvrsn=baf81d52_4.

